# Overoxidation of chloroplast 2-Cys peroxiredoxins: balancing toxic and signaling activities of hydrogen peroxide

**DOI:** 10.3389/fpls.2013.00310

**Published:** 2013-08-19

**Authors:** Leonor Puerto-Galán, Juan M. Pérez-Ruiz, Julia Ferrández, Beatriz Cano, Belén Naranjo, Victoria A. Nájera, Maricruz González, Anna M. Lindahl, Francisco J. Cejudo

**Affiliations:** ^1^Instituto de Bioquímica Vegetal y Fotosíntesis, Universidad de SevillaSevilla, Spain; ^2^Consejo Superior de Investigaciones CientíficasSevilla, Spain

**Keywords:** chloroplast, hydrogen peroxide, peroxiredoxin, redox regulation, thioredoxin, oxidative stress

## Abstract

Photosynthesis, the primary source of biomass and oxygen into the biosphere, involves the transport of electrons in the presence of oxygen and, therefore, chloroplasts constitute an important source of reactive oxygen species, including hydrogen peroxide. If accumulated at high level, hydrogen peroxide may exert a toxic effect; however, it is as well an important second messenger. In order to balance the toxic and signaling activities of hydrogen peroxide its level has to be tightly controlled. To this end, chloroplasts are equipped with different antioxidant systems such as 2-Cys peroxiredoxins (2-Cys Prxs), thiol-based peroxidases able to reduce hydrogen and organic peroxides. At high peroxide concentrations the peroxidase function of 2-Cys Prxs may become inactivated through a process of overoxidation. This inactivation has been proposed to explain the signaling function of hydrogen peroxide in eukaryotes, whereas in prokaryotes, the 2-Cys Prxs of which were considered to be insensitive to overoxidation, the signaling activity of hydrogen peroxide is less relevant. Here we discuss the current knowledge about the mechanisms controlling 2-Cys Prx overoxidation in chloroplasts, organelles with an important signaling function in plants. Given the prokaryotic origin of chloroplasts, we discuss the occurrence of 2-Cys Prx overoxidation in cyanobacteria with the aim of identifying similarities between chloroplasts and their ancestors regarding their response to hydrogen peroxide.

## INTRODUCTION

Oxygenic photosynthesis is an essential process for life on Earth because it allows the use of light and water to produce biomass and oxygen. However, it is also a process potentially harmful due to the transport of electrons in the presence of oxygen, which inevitably produces reactive oxygen species (ROS). Several environmental challenges such as drought, low or high temperature, high light intensity, or salinity, alter chloroplast ROS homeostasis producing oxidative stress (Miller et al., 2010). To adequately respond to these stressful conditions chloroplasts are equipped with different antioxidant systems both enzymatic and non-enzymatic. It should be taken into account that besides their harmful effect, ROS have also signaling function (Laloi et al., 2007). This is the case of hydrogen peroxide, which is produced at high rate in chloroplasts of photosynthetic cells and has an important signaling activity (Mubarakshina et al., 2010), as confirmed by genome-wide expression analyses in tobacco and *Arabidopsis* (Vandenabeele et al., 2003; Vanderauwera et al., 2005).

Peroxiredoxins (Prxs), thiol-based peroxidases able to reduce hydrogen peroxide, peroxynitrite and organic peroxides, are among the most abundant chloroplast enzymatic antioxidant systems. Prxs are universally present in any type of organisms from bacteria to animals and plants (Dietz, 2003, 2011; Wood et al., 2003a; Rhee et al., 2005; Hall et al., 2009). These enzymes are classified into different classes including typical 2-Cys Prxs, which are homodimeric, atypical 2-Cys Prxs, which are monomeric, and 1-Cys Prxs. Both typical and atypical 2-Cys Prxs share a similar reaction mechanism involving two conserved Cys residues, termed peroxidatic and resolving, respectively (Wood et al., 2003a; Hall et al., 2009). During the catalytic cycle the peroxidatic Cys becomes transiently oxidized as sulfenic acid (-SOH) and then condenses with the resolving Cys to form a disulfide bridge. In the case of the typical 2-Cys Prxs, which are dimeric, the enzyme is fully oxidized when both pairs of catalytic Cys residues (peroxidatic and resolving) form disulfides, which have to be reduced to initiate a new catalytic cycle. This reduction is performed by a thiol-oxidoreductase, which usually is thioredoxin (Trx), though glutaredoxin and cyclophilins are also able to participate in this step. At high peroxide concentrations, the sulfenic acid intermediate of the peroxidatic Cys may become overoxidized to sulfinic (-SO_2_H) or even sulfonic (-SO_3_H) acids, which causes the inactivation of the enzyme. Based on the different sensitivities of 2-Cys Prxs to overoxidation, which is higher in enzymes from eukaryotes than from prokaryotes, Wood et al. (2003b) proposed the floodgate hypothesis. According to this hypothesis, oxidizing conditions promote the inactivation by overoxidation of sensitive 2-Cys Prxs in eukaryotic organisms, provoking a transient further increase of hydrogen peroxide, which may then be used as second messenger (Wood et al., 2003b). In contrast, in prokaryotic organisms, the 2-Cys Prxs of which were considered to be insensitive, hydrogen peroxide is efficiently reduced and does not accumulate, thus having a less important function in signaling. Different reports confirm the relevant role of the hydrogen peroxide-dependent inactivation of 2-Cys Prxs in signaling processes in eukaryotic organisms (Karplus and Poole, 2012; Rhee et al., 2012). A notion reinforced by the recent finding that 2-Cys Prx overoxidation is a conserved marker of circadian rhythmicity (Edgar et al., 2012).

In plants, Prxs are encoded by a gene family, which in *Arabidopsis* is composed of ten members (Dietz, 2003). The first plant Prx identified was a 1-Cys Prx highly expressed in barley grains (Stacy et al., 1996). Later it was shown that this 1-Cys Prx accumulates in the nucleus of cereal seed tissues that undergo intense oxidative stress (Stacy et al., 1999; Pulido et al., 2009), suggesting a function in the antioxidant protection of nuclear structures. Chloroplasts are the organelles with the highest content of Prxs. The *Arabidopsis* chloroplast contains two almost identical typical 2-Cys Prxs, termed A and B, and atypical monomeric Prxs Q and IIE (Dietz, 2003, 2011). Although 2-Cys Prxs are among the most abundant plastidial proteins, a double mutant of *Arabidopsis*, which is a severe knock down for 2-Cys Prxs, shows a surprisingly mild phenotype (Pulido et al., 2010) suggesting that other antioxidant systems, such as the ascorbate-glutathione cycle in combination with superoxide dismutase, are able to compensate for 2-Cys Prx deficiency. Moreover, it was shown that chloroplast 2-Cys Prxs are sensitive to overoxidation, hence behaving as eukaryotic-type enzymes despite the endosymbiont origin of this organelle (Kirchsteiger et al., 2009). A more in-depth analysis of 2-Cys Prx in cyanobacteria showed that the enzyme from *Anabaena* is more sensitive to overoxidation than the *Synechocystis* enzyme (Pascual et al., 2010).

In this review we will discuss our present knowledge of the mechanisms controlling 2-Cys Prxs reduction and overoxidation in chloroplasts with emphasis in the effect of the redox status of 2-Cys Prxs on the activity of these enzymes. Moreover, we will discuss how the redox status of the chloroplast influences the signaling function of this organelle, which is essential to harmonize the growth and development of the different plant organs.

## THE PATHWAYS OF 2-Cys Prxs REDUCTION IN CHLOROPLASTS

The *Arabidopsis* chloroplast is equipped with two almost identical typical 2-Cys Prxs, A and B, and atypical Prxs Q and IIE (Dietz et al., 2006). Although all these enzymes are relatively abundant, 2-Cys Prxs are among the most abundant proteins of the chloroplast (Dietz, 2011). Concerning their suborganellar localization, the presence of Prx IIE in the chloroplast stroma has been established, though its interaction with internal membranes was not analyzed (Bréhélin et al., 2003). Prx Q, which was described initially as associated to thylakoids (Lamkemeyer et al., 2006), was later localized in the thylakoid lumen (Petersson et al., 2006). Therefore, the exact localization of Prx Q still awaits confirmation (Dietz, 2011). 2-Cys Prxs A and B are localized in the chloroplast stroma in dimeric form, but become associated to the thylakoid membrane in their oligomeric form (Konig et al., 2002).

The function of the chloroplast-localized Prxs was first addressed by the generation of transgenic plants with antisense suppression of 2-Cys Prx expression (Baier et al., 2000), and then by the analysis of *Arabidopsis* mutants (Pulido et al., 2010). Single mutants with reduced levels of 2-Cys Prx A or lacking 2-Cys Prx B showed no phenotypic differences as compared with wild type plants, suggesting redundant functions of these enzymes (Pulido et al., 2010). Surprisingly, the double mutant Δ*2cp*, which is knock out for 2-Cys Prx B and a severe knock down for 2-Cys Prx A, shows almost wild type phenotype (Pulido et al., 2010). However, it was not possible to obtain a double knock out mutant, which suggests that plants cannot survive without at least a small amount of chloroplast 2-Cys Prxs. Most probably this is not exclusively due to their peroxidase activity, which can be compensated for by the other antioxidant systems of the chloroplast, such as the ascorbate-glutathione cycle in combination with superoxide dismutase. 2-Cys Prxs are complex enzymes showing different activities associated with different quaternary structures. As initially shown for the enzyme from yeast (Jang et al., 2004), the low-molecular-weight (LMW) form of 2-Cys Prxs shows predominantly peroxidase activity, whereas higher molecular weight (HMW) forms lack peroxidase activity while gaining chaperone activity. Interestingly, the switch from LMW to HMW is triggered under conditions of oxidative stress.

**FIGURE 1:**
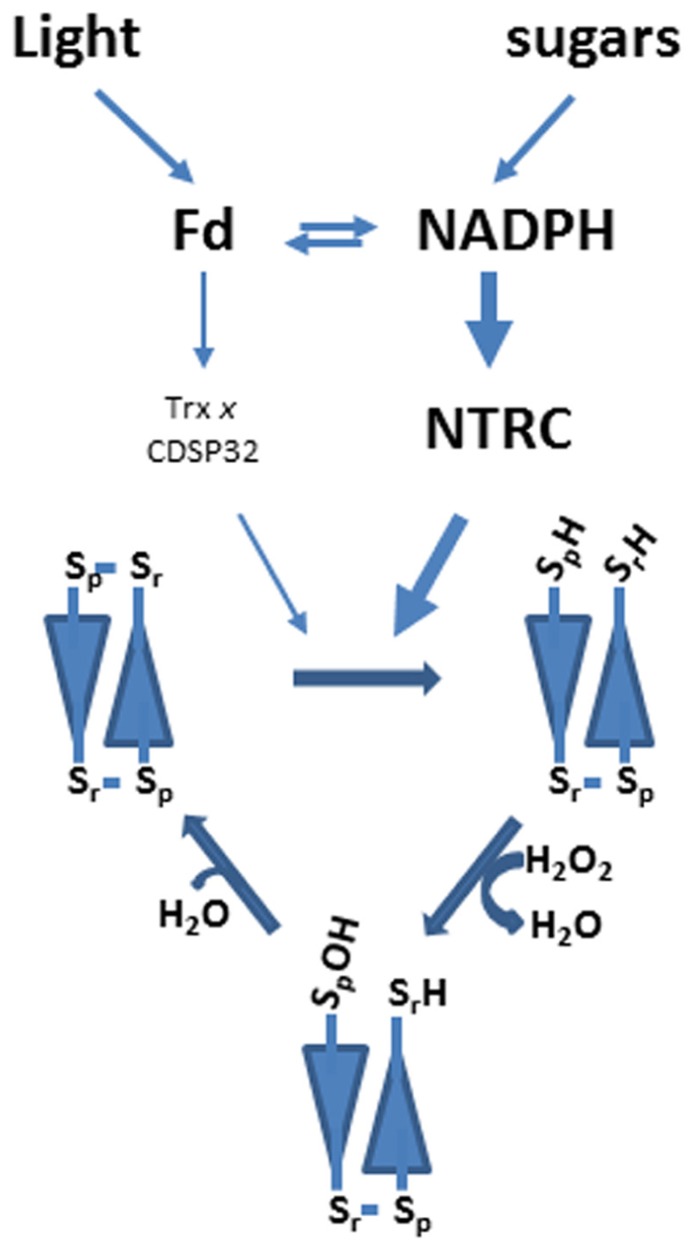
**Reaction mechanism and pathways for 2-Cys Prx reduction in chloroplasts.** Typical 2-Cys Prxs are homodimeric enzymes arranged in head-to-tail conformation. Catalysis is performed by two cysteine residues, peroxidatic (*S*_p_) and resolving (*S*_r_). The peroxidatic cysteine reacts with hydrogen peroxide producing a molecule of water and becoming oxidized as sulfenic acid. In a second step the resolving cysteine condenses with the sulfenic acid intermediate so that a molecule of water is produced and both cysteines are linked by a disulfide bridge. For a new catalytic cycle the disulfide has to be reduced. In plant chloroplasts both *in vitro* and *in vivo* analyses suggest that NTRC is more relevant for 2-Cys Prx reduction than other plastidial Trxs such as CDSP32 and Trx *x*.

Chloroplast 2-Cys Prxs have a reaction mechanism similar to the enzyme from other eukaryotic organisms. The LMW form of the enzyme is arranged as a head-to-tail homodimer, which in its reduced form displays peroxidase activity (**Figure 1**). Therefore, 2-Cys Prxs can be considered as symmetric enzymes having two identical active sites. The catalytic cycle is initiated by the attack of the peroxidatic Cys to the peroxide rendering the corresponding alcohol, or water in the case of hydrogen peroxide, and the Cys residue oxidized to sulfenic acid (**Figure 1**). In a second step, the sulfenic acid intermediate is condensed with the resolving Cys producing a molecule of water and both Cys linked by a disulfide bridge (**Figure 1**). For a new catalytic cycle this disulfide has to be reduced. In chloroplasts Broin et al. (2002) proposed that a previously identified protein, termed CDSP32, which is formed by two Trx folds, with only one of them harboring a Trx active site, acted as reductant of 2-Cys Prxs. The *in vitro *analysis of several plastidial Trxs led Collin et al. (2003) to propose Trx *x* as the most efficient reductant of these enzymes. Finally, the chloroplast localized NADPH-dependent thioredoxin reductase C (NTRC), a peculiar NTR with a joint Trx domain at the C-terminus (Serrato et al., 2002, 2004) was shown to combine both NTR and Trx activity to efficiently reduce plastidial 2-Cys Prx (Moon et al., 2006; Pérez-Ruiz et al., 2006; Alkhalfioui et al., 2007). The notion that NTRC is the most efficient reductant of chloroplast 2-Cys Prxs was subsequently confirmed in further studies by *in vivo* analysis, based on fluorescence resonance energy transfer (FRET) assays, which showed interaction of 2-Cys Prx with NTRC but not with Trx *x* (Muthuramalingam et al., 2009). In addition, the redox status of 2-Cys Prxs was similar in wild type and Trx *x* knock out mutant plants, whereas the *ntrc* mutant showed a severely impaired redox status (Pulido et al., 2010). In summary, as depicted in **Figure 1**, chloroplast 2-Cys Prxs have a mode of action similar to the enzymes from other eukaryotic organisms and is predominantly reduced by NTRC. The required reducing power in form of NADPH is produced either by the photosynthetic electron transport chain, which occurs during the day, or from sugars by the initial reactions of the oxidative pentose phosphate pathway, which would be the predominant pathway during the night (Spínola et al., 2008; Cejudo et al., 2012). Though not experimentally established, it is expected that Trx *x* and CDSP32 use the reducing power of reduced ferredoxin (Fd) in a reaction catalyzed by Fd-dependent Trx reductase (FTR). Results from our group show that NTRC is unable to reduce plastidial Trxs, such as Trx *x* or CDSP32 (Pérez-Ruiz et al., 2006; Bernal-Bayard et al., 2012). Therefore, it seems that the two different pathways, NTRC and FTR/Trx, for 2-Cys Prx reduction are not connected.

## CHLOROPLAST 2-Cys Prxs ARE SENSITIVE TO OVEROXIDATION

**FIGURE 2:**
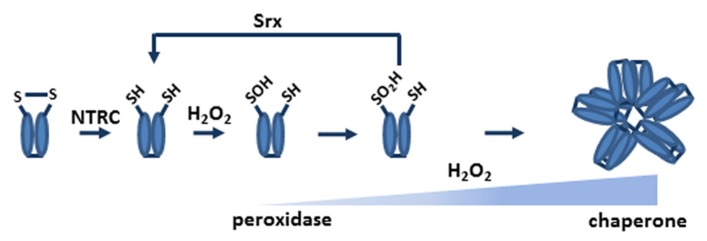
**NTRC and Srx determine the redox status of chloroplast 2-Cys Prxs.** Under oxidant conditions, the sulfenic acid intermediate of the peroxidatic cysteine residue may be further oxidized to sulfinic acid. The reduction of the enzyme, which is most efficiently performed by NTRC, is a pre-requisite for sulfenic acid formation and, thus, for overoxidation. Srx is able to catalyze the reversion of the overoxidized to the reduced form of the enzyme. Therefore, the redox status of chloroplast 2-Cys Prxs is highly dependent of NTRC and Srx. The quaternary structure of 2-Cys Prxs determines the activity of these enzymes. In the reduced form the enzyme is a dimer and shows peroxidase activity; overoxidation favors the formation of the decameric form, which lacks peroxidase activity and shows chaperone activity.

The study of the reaction mechanism of chloroplast 2-Cys Prxs revealed that the enzyme may become irreversibly oxidized during the catalytic cycle and shows tendency to form oligomers (Konig et al., 2002). These properties of chloroplast 2-Cys Prxs gained interest when Wood et al. (2003b) proposed the floodgate hypothesis according to which the signaling function of hydrogen peroxide in eukaryotic organisms is due to the overoxidation of the peroxidatic cysteine at the active site of 2-Cys Prxs. As mentioned above, during catalysis the peroxidatic cysteine becomes transiently oxidized to sulfenic acid, which under oxidizing conditions may be overoxidized to sulfinic or even sulfonic acid (**Figure 2**). This overoxidation inhibits the peroxidase activity of the enzyme thus allowing the local accumulation of hydrogen peroxide, which exerts its function as second messenger (Wood et al., 2003b). Though initially it was thought that overoxidation was an irreversible process, it was then found that sulfiredoxin (Srx) is able to reverse the overoxidized form to the reduced form of the enzyme in a reaction that required ATP and Mg^2^^+^ (Biteau et al., 2003; Woo et al., 2003). Overoxidation favors the formation of the HMW form of 2-Cys Prxs, which promotes the chaperone activity of these enzymes (**Figure 2**). All these data, obtained from analyses with yeast and human enzymes, indicated that the redox status of 2-Cys Prxs is essential to determine their peroxidase or chaperone activity, making them efficient sensors and key components of the response to oxidant conditions (Karplus and Poole, 2012).

In plants, the chloroplast is an essential organelle not only because of photosynthesis, but also because it is the site of synthesis of a variety of compounds, such as hormones, which play a role in signaling. The role of the chloroplast as an important source of hydrogen peroxide is well known (Mubarakshina et al., 2010). Indeed, we have recently shown that restitution of the redox homeostasis exclusively in chloroplasts, by expressing NTRC in the *ntrc* background mutant under the *RbcS* promoter, was necessary and sufficient to recover wild type growth and development of lateral roots regardless of the impaired redox homeostasis in root amyloplasts (Ferrández et al., 2012; Kirchsteiger et al., 2012). Therefore, whether or not chloroplast 2-Cys Prxs undergo overoxidation and the mechanisms controlling the redox status of the enzyme are relevant questions to determine their antioxidant and/or signaling function.

Two-dimensional gel electrophoresis analysis of 2-Cys Prx from wild type and mutants deficient in either 2-Cys Prx A or 2-Cys Prx B from *Arabidopsis* revealed the overoxidation of both enzymes (Kirchsteiger et al., 2009). Surprisingly, the NTRC knock out mutant showed lower level of 2-Cys Prx overoxidation than wild type plants, despite the fact that the deficiency of NTRC may cause oxidative stress. This was a first indication suggesting that the reduction of the enzyme, as a pre-requisite for the formation of the sulfenic acid intermediate, is required for the subsequent overoxidation to sulfinic acid, as outlined in **Figure 2**. The other component affecting the level of 2-Cys Prx overoxidation in chloroplasts is Srx, which is encoded in plants by a single gene, the protein showing dual targeting to chloroplast and mitochondria (Liu et al., 2006; Iglesias-Baena et al., 2011). Chloroplast Srx was shown to effectively reverse 2-Cys Prx overoxidation (Rey et al., 2007; Iglesias-Baena et al., 2010), though enzyme was also shown to have redox-independent nuclease activity (Chi et al., 2012). The analysis of an *Arabidopsis* Srx knock out mutant revealed a function of the enzyme in the response to photooxidative stress (Rey et al., 2007). In addition, it was shown that the overoxidation of the chloroplast 2-Cys Prxs, like those of other eukaryotic organisms, triggers the oligomerization of the enzyme, which diminishes the peroxidatic activity while it increases the chaperone activity (Barranco-Medina et al., 2009).

Factors affecting 2-Cys Prx overoxidation in chloroplasts are summarized in the scheme outlined in **Figure 2**. The reduction of the enzyme, which is predominantly performed by NTRC, is a pre-requisite for the formation of the sulfenic acid intermediate. At high peroxide concentrations this intermediate may become increasingly overoxidized, which switches the peroxidase to chaperone activity of the enzyme. Reversion of the overoxidized form of 2-Cys Prx is catalyzed by Srx in a reaction that requires ATP and Mg^2+^. According to this scheme, two enzymes, NTRC and Srx, seem to play a central role in controlling the redox status of 2-Cys Prxs in chloroplasts. It has been proposed that 2-Cys Prx may exert a critical function by balancing antioxidant and signaling activities of chloroplast produced hydrogen peroxide (Dietz et al., 2006). This function is probably essential as suggested by the fact that the double knock out mutant lacking both 2-Cys Prx A and B seems not viable. Nevertheless, much effort is still required to determine the reason why these enzymes have such an essential function for plant survival.

## THE CYANOBACTERIAL ORIGIN OF CHLOROPLAST 2-Cys Prx OVEROXIDATION

According to the floodgate hypothesis, the signaling activity of hydrogen peroxide in eukaryotic organisms is based on the inactivation of 2-Cys Prxs by overoxidation, which allows the transient increase in the peroxide necessary to act as second messenger (Wood et al., 2003b). Structural analysis identified the GG(L/V/I)G and YF motifs in sensitive enzymes, and established that the peroxidatic cysteine is 14 Å away from the resolving cysteine, which makes the eukaryotic enzymes about 100-fold more sensitive to overoxidation than the prokaryotic ones (Wood et al., 2003b). Chloroplast 2-Cys Prxs are sensitive to overoxidation (Broin and Rey, 2003; Kirchsteiger et al., 2009; Iglesias-Baena et al., 2010), thus behaving as expected for enzymes of a eukaryotic organelle. Because it is well established that chloroplasts evolved from a prokaryotic endosymbiont (Gould et al., 2008), it arises the question whether 2-Cys Prx sensitivity was already present in the prokaryotic endosymbiont or was a gain-of-function of these enzymes that occurred during chloroplast evolution. To address this question, Pascual et al. (2010) analyzed the presence of the GG(L/V/I)G and YF motifs in the genes encoding 2-Cys Prxs from different sources. This search confirmed the presence of sensitive 2-Cys Prxs, characterized by the presence of both motifs, in eukaryotes. However, it revealed an unexpectedly large number of 2-Cys Prx from prokaryotic organisms containing the GG(L/V/I)G and YF motifs, thus being putatively sensitive to overoxidation. Interestingly, the 2-Cys Prxs from several cyanobacteria, such as *Anabaena* sp. PCC7120 and *Synechocystis* sp. PCC6803, were found to contain these motifs. Biochemical analyses revealed that 2-Cys Prx from *Anabaena* sp. PCC7120 shows a level of sensitivity to overoxidation similar to that of the chloroplast enzymes, whereas 2-Cys Prx from *Synechocystis* sp. PCC6803 is less sensitive (Pascual et al., 2010). Moreover, *in vivo* analyses showed different strategies of these cyanobacterial strains to respond to oxidative stress. While *Anabaena* showed high sensitivity, *Synechocystis* survived higher concentrations of hydrogen peroxide. The strategy based on high efficiency of hydrogen peroxide detoxification provides higher resistance though, as it is rapidly reduced, the peroxide cannot be used for signaling. In contrast, the *Anabaena* strategy, based on low capacity of detoxification, causes the increase of hydrogen peroxide required to act as second messenger, though it may have as well a harmful effect. Interestingly, the strategy of chloroplasts, which are equipped with sensitive 2-Cys Prxs and lack catalase, is very similar to the *Anabaena *strategy. This is in agreement with the proposal that chloroplasts originated from cyanobacterial strains similar to present day *Anabaena *species (Deusch et al., 2008).

## CONCLUDING REMARKS AND FUTURE PROSPECTS

The inactivation of the peroxidase activity of 2-Cys Prxs, caused by the overoxidation of their peroxidatic cysteines, has been proposed to be essential for the signaling function of hydrogen peroxide in eukaryotic organisms. In chloroplasts, which constitute an important source of hydrogen peroxide and have a prominent signaling function in plants, 2-Cys Prxs are among the most abundant proteins. Despite the prokaryotic origin of the plant chloroplast, the 2-Cys Prxs of this organelle undergo peroxide-mediated overoxidation, thus behaving as eukaryotic-type enzymes. The redox status of chloroplast 2-Cys Prxs, mostly controlled by NTRC and Srx, may balance the antioxidant and signaling functions of chloroplast-produced hydrogen peroxide and, thus, its activity as second messenger. Although much progress has been made on the biochemical properties of 2-Cys Prxs, little is yet known about the mechanisms explaining their function in signaling. The identification of the targets of these enzymes may be of aid to establish these functions.

## Conflict of Interest Statement

The authors declare that the research was conducted in the absence of any commercial or financial relationships that could be construed as a potential conflict of interest.
